# Liraglutide-induced Acute Gastroparesis

**DOI:** 10.7759/cureus.3791

**Published:** 2018-12-28

**Authors:** Puja Rai, Mahmoud Y Madi, Aaron Dickstein

**Affiliations:** 1 Internal Medicine, Tufts Medical Center, Boston, USA; 2 Gastroenterology, Tufts Medical Center, Boston, USA

**Keywords:** gastroparesis, gastric outlet obstruction, liraglutide

## Abstract

We describe a case of liraglutide-induced acute gastroparesis in a 52-year-old man with a history of well-controlled type 2 diabetes who presented with symptoms of gastric outlet obstruction. The patient responded markedly to conservative treatment with gastric suctioning, antiemetic and prokinetic therapy, and discontinuation of liraglutide with a resolution of his symptoms. This case highlights the importance of considering drug-induced gastroparesis as an etiology of unexplained upper abdominal pain, nausea, and early satiety, especially in the absence of mechanical obstruction.

## Introduction

Gastroparesis is a syndrome of delayed gastric emptying without mechanical obstruction. The condition typically causes nausea, vomiting, early satiety, postprandial fullness, bloating, and/or upper abdominal pain [1]. Common etiologies include diabetes mellitus, post-surgical, Parkinson’s disease, and medication-induced. Approximately one-third of cases are idiopathic in origin. The diagnosis is made by documenting upper gastrointestinal symptoms, excluding mechanical obstruction, and demonstrating delayed gastric emptying. Treatment is targeted at reducing symptoms, nutritional support, diet modification, improvement of gastric emptying with prokinetics, and correcting the precipitating cause, if possible [2].

## Case presentation

**Figure 1:**
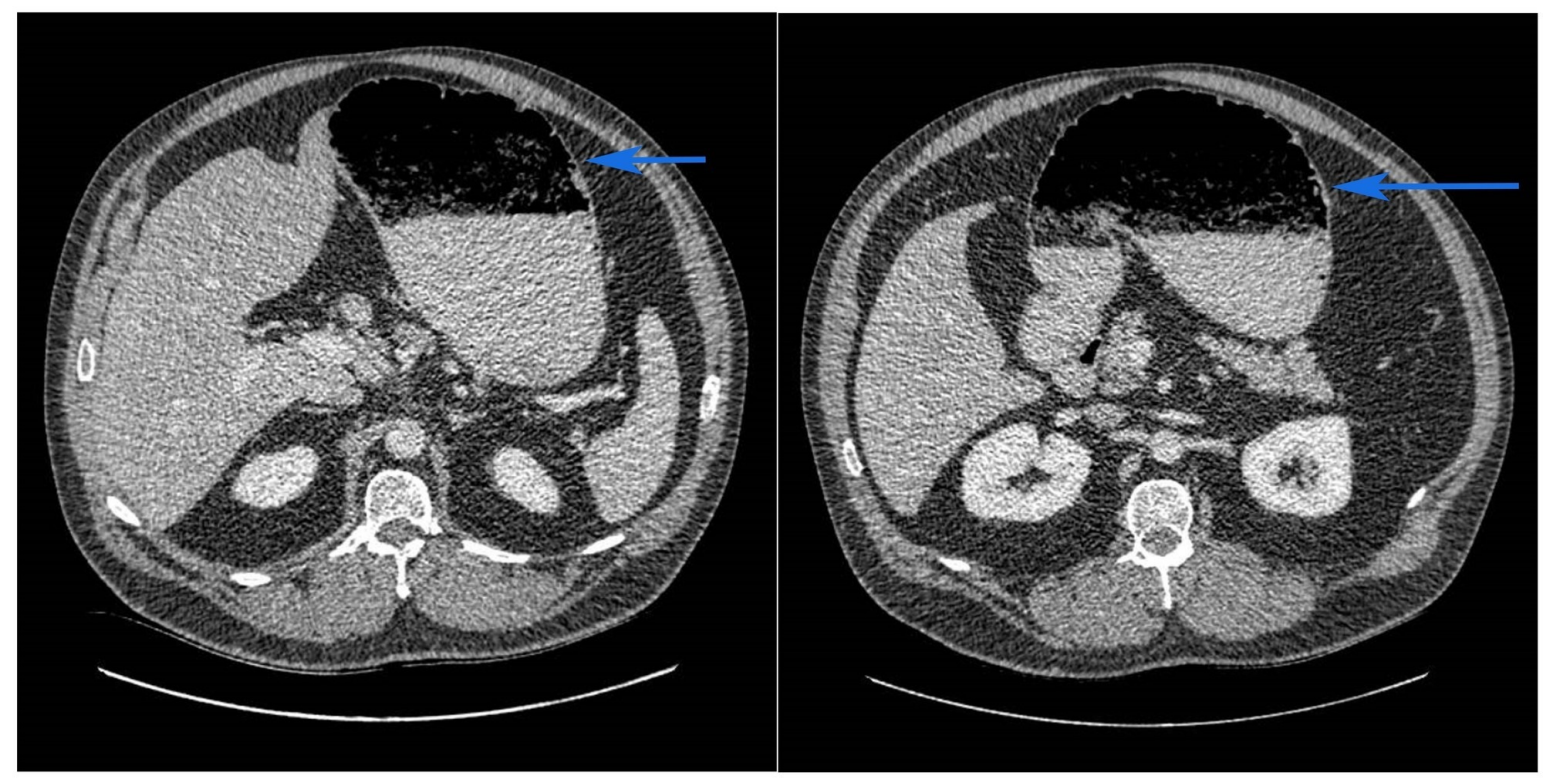
Computed tomography (CT) scan of the abdomen CT scan of the abdomen showing a markedly distended stomach (blue arrow) with food/debris and normal caliber duodenum without an obvious lesion

A 52-year-old man with a past medical history of hypertension, hyperlipidemia and well-controlled type 2 diabetes with no prior history of gastroparesis presented with nausea, abdominal distension, and pain of one-week duration. The patient initially reported symptoms of early satiety and excessive bloating, leading to nausea and progressive abdominal distension. He then developed more acute, severe epigastric and left upper quadrant pain. Upon arrival at a local emergency department, a nasogastric tube was placed with over 1 liter of fluids suctioned. This provided instant symptom relief but raised concern for gastric outlet obstruction. An abdominal CT scan at the time revealed markedly distended stomach with food/debris and normal caliber duodenum without an obvious lesion (Figure 1). Due to concern for gastric outlet obstruction and the possible need for surgery, the patient was transferred to our tertiary care center for further evaluation. Physical examination on transfer was notable for a soft but mildly distended abdomen with mild tenderness to palpation in the epigastrium and left upper quadrant with faint bowel sounds and negative succussion splash. His nasogastric tube was still putting out significant fluid to suction. Laboratory testing revealed glucose level of 105 mg/dL, hemoglobin A1c of 7.0, normal liver function tests, normal lipase, normal CBC and chemistry. Abdominal x-ray showed a non-obstructive gas pattern with no intestinal dilatation. On review of his medications, he mentioned a recent start of liraglutide at 1.2 mg subcutaneously daily for optimization of his glycemic control. He was not taking any opiates prior to and during his hospital stay.

**Figure 2:**
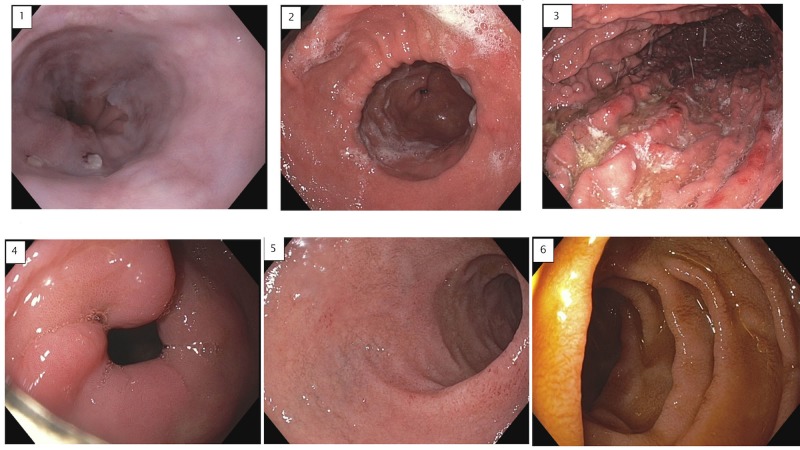
Esophagogastroduodenoscopy (EGD) Series of images obtained during EGD showing: 1) normal gastroesophageal junction; 2) gastric antrum with no mass lesions; 3) gastric body with evidence of mild gastritis; 4) normal pylorus with no mass lesions or ulcers; 5) normal duodenal bulb; 6) normal second part of the duodenum.

Shortly after admission, he underwent an upper endoscopy, which showed no evidence of an obstructing lesion, tumor, or bezoar (Figure 2). The pylorus was patent and easily traversed. There was mild irritation in the gastric body, likely related to nasogastric tube trauma.

Given the temporal relationship of his symptoms to the recent initiation of liraglutide, this was determined to be the likely etiology of his acute presentation. The esophagogastroduodenoscopy (EGD) was helpful to exclude a mechanical obstruction. Notably, by withholding further doses of liraglutide, dietary modification, and a brief course of antiemetics and metoclopramide, his symptoms completely resolved.

## Discussion

Gastroparesis is a chronic disorder manifested by delayed gastric emptying without evidence of mechanical obstruction. In the United States, approximately 5 million patients suffer from some form of gastroparesis [3]. Nausea and vomiting are the two most prevalent symptoms. Abdominal pain is also common and was reported in up to two-thirds of all patients [4-5].   

In addition to the common etiologies mentioned earlier, rarer causes include certain neurological diseases, amyloidosis, and connective tissue disorders [1]. Many cases remain idiopathic after workup.

Symptoms of gastroparesis are nonspecific, as the condition can mimic dyspepsia, peptic ulcer disease, or gastric or small bowel obstruction [1]. In suspected gastroparesis, an upper endoscopy is indicated to exclude a mechanical obstruction. In addition, a four-hour solid-phase gastric emptying scintigraphy test to assess gastric retention is recommended to confirm the diagnosis [6]. In our patient, we did not formally test for gastroparesis, given his marked improvement with the discontinuation of liraglutide and his lack of symptoms prior to the initiation of the drug.

The pathophysiological mechanism of gastroparesis is not fully understood but involves abnormalities in multiple interacting cell types, including the extrinsic nervous system, enteric nervous system, interstitial cells of Cajal, smooth muscles and immune cells [7]. Other contributing factors include acute fluctuations in blood glucose, medication side effects, and psychosomatic factors, such as pain through autonomic mechanisms [6].

Liraglutide is a long-acting GLP-1 analog that activates the GLP-1 receptor in pancreatic beta cells to stimulate glucose-dependent insulin secretion while reducing inappropriate glucagon secretion from pancreatic alpha cells. Liraglutide also suppresses gastric emptying, leading to decreased food intake as a result of relaxation of the proximal stomach accompanied by increased tonic contraction of the antropyloric region [8]. Subcutaneous injections at doses up to 1.8 mg are administered once-daily, while higher doses can be used for chronic weight management [9]. Given that GLP-1 is known to slow gastric emptying and cause gastrointestinal (GI) side effects, the initial recommended dosing is 0.6 mg once daily for one week with up-titration as tolerated for effective glycemic control. While gastroparesis is a labeled side-effect of liraglutide, few reports have been documented in the literature. Severe acute gastroparesis presenting with significant pain and gastric-outlet obstruction-like syndrome has not been reported previously. In one study, patients were randomized to liraglutide 1.8 mg, liraglutide 3.0 mg, and placebo with gastric emptying testing. Five-hour gastric emptying was equivalent between groups, although reductions in one-hour gastric testing of 23% in liraglutide 3.0 mg and 13% with 1.8 mg versus placebo were observed [9]. Another study demonstrated that liraglutide 3.0 mg delays gastric emptying at five weeks and 16 weeks compared to effects of placebo, with slower gastric emptying at five weeks relative to 16 weeks [10]. Therefore, in patients found to have gastroparesis, especially in those recently initiated on liraglutide, the medication should be considered as a potential culprit.

The management of gastroparesis predominantly focuses on symptom control. Initial therapy involves correction of fluid, electrolyte, and nutritional deficiencies. Once improved, patients are advised on dietary modification, including small frequent meals with limited fat and fiber content [5, 7]. If oral intake is inadequate, enteral feeding to bypass the dysfunctional stomach should be considered [7]. Prokinetic medications are often effective in reducing symptoms of nausea and vomiting. Moreover, optimal glycemic control is critical to minimize the inhibitory effects of hyperglycemia on gastric emptying [1, 5]. In addition to the above, a review of current medications can often provide a critical clue to the trigger for symptoms. Our case reinforces this last point; physicians should be cautious when prescribing GLP-1 analogs in diabetics as they slow gastric emptying and may rarely lead to acute symptoms of gastroparesis. 

## Conclusions

Drug-induced gastroparesis, specifically with liraglutide use, should be considered in patients presenting with significant abdominal distension, pain, and nausea once mechanical obstruction has been ruled out. It should be suspected in diabetic patients with recent liraglutide initiation, especially at higher doses. Management includes discontinuation of the offending medication, symptom control with antiemetics and prokinetic agents, nutritional support, and dietary modification.
